# Distribution of Adiponectin Receptors in the Brain of Adult Mouse: Effect of a Single Dose of the Adiponectin Receptor Agonist, AdipoRON, on Ischemic Stroke

**DOI:** 10.3390/brainsci12050680

**Published:** 2022-05-23

**Authors:** Julien Clain, David Couret, Cynthia Planesse, Pascale Krejbich-Trotot, Olivier Meilhac, Christian Lefebvre d’Hellencourt, Wildriss Viranaicken, Nicolas Diotel

**Affiliations:** 1 Université de la Réunion, INSERM, UMR 1188 Diabète Athérothrombose Thérapies Réunion Océan Indien (DéTROI), Plateforme CYROI, 97490 Sainte-Clotilde, France; julien.clain@univ-reunion.fr (J.C.); david.couret@chu-reunion.fr (D.C.); cynthia.planesse@univ-reunion.fr (C.P.); olivier.meilhac@inserm.fr (O.M.); christian.lefebvre-d-hellencourt@univ-reunion.fr (C.L.d.); 2 CHU de La Réunion, 97400 Saint-Denis, France; 3 Processus Infectieux en Milieu Insulaire Tropical (PIMIT), INSERM, UMR 1187, CNRS UMR9192, IRD UMR249, Université de La Réunion, 94791 Sainte-Clotilde, France; pascale.krejbich@univ-reunion.fr

**Keywords:** AdipoRON, adiponectin, AdipoR, MCAO, infarct, neuroprotection, stroke

## Abstract

Adiponectin exhibits pleiotropic effects, including anti-inflammatory, anti-apoptotic, anti-oxidant, and neuroprotective ones. Although some studies have documented brain expression in different rodent models of its receptors, AdipoR1 and AdipoR2, their global distribution remains incomplete. Here, we demonstrated that both AdipoR are widely distributed in the brains of adult mice. Furthermore, by double immunostaining studies, we showed that AdipoR1 and AdipoR2 are mainly expressed in neurons and blood vessels. Then, considering the wide distribution of both receptors and the neuroprotective effects of adiponectin, we tested the therapeutic effect of a single injection of the adiponectin receptor agonist, AdipoRON (5 mg.kg^−1^), 24 h after stroke in a model of middle cerebral artery occlusion technique (MCAO). Under our experimental conditions, we demonstrated that AdipoRON did not modulate the infarct volume, cell death, neuroinflammatory parameters including microglia activation and oxidative stress. This study suggests that a protocol based on multiple injections of AdipoRON at a higher dose after MCAO could be considered to promote the therapeutic properties of AdipoRON on the brain repair mechanism and recovery.

## 1. Introduction

The white adipose tissue recently emerged as an endocrine organ producing many hormonal factors called adipokines. Adiponectin (Acrp30 or AdipoQ) is one of the most abundant adipokines in human blood (2–20 µg.mL^−1^) [1]. This hormone is implied in both lipid and glucose metabolism, regulating among others, insulin and blood glucose levels [2,3,4]. Several forms of adiponectin can be detected in the blood: a monomeric globular form, low molecular weight trimers, medium molecular weight hexamers and high molecular weight multimers [1]. The adiponectin hormone mainly binds two membrane receptors: AdipoR1 and AdipoR2 [5,6].

Through its receptors, adiponectin exerts pleiotropic effects that are mainly cyto-protective including antioxidant, anti-inflammatory and anti-apoptotic ones [7,8,9,10]. It also exhibits protective properties considering the cardiovascular and renal systems [10,11,12,13]. The diversity of the effects mediated by adiponectin is supported by the variety of adiponectin forms and the tissue- and cell-specific expression of its receptors [14].

Expression of adiponectin receptors is not restricted to the periphery (i.e., muscle, liver, adipose tissue) but is also found in the central nervous system (CNS). AdipoR1 and AdipoR2 are expressed in several encephalic regions including the cerebral cortex, the hypothalamus, the pituitary gland, the brainstem and the hippocampus [1,15,16]. Recent data obtained by in situ hybridization and RNA sequencing of different cell types within the cerebral cortex of mice have also demonstrated that both *AdipoR1* and *AdipoR2* transcripts are detected in neurons, immature and mature oligodendrocytes, microglia, astrocytes and endothelial cells [17,18]. Adiponectin receptor expression is also observed in the main adult neurogenic niches: the dentate gyrus [19] and the subventricular zone of the lateral ventricle (SVZ) [18,20]. Despite these different studies, the general overall distribution of AdipoR1 and AdipoR2 proteins remains unclear in the CNS. Indeed, most studies conducted focus on a single brain region, at a different stage of development, in different models (i.e., mice vs. rat) and also use different techniques (i.e., PCR, in situ hybridization, or immunostaining with different antibodies). So far, there is not a complete overview of AdipoR distribution in the brain of mice at one stage of development. Nevertheless, all these studies demonstrate AdipoR expression within the encephalon, strongly suggesting that adiponectin may play roles beyond the metabolic sphere.

Indeed, adiponectin displays interesting properties participating in the establishment of the blood-brain barrier [21]. It is thought to promote cognitive function through the modulation of hippocampal plasticity [19] and to have anti-inflammatory and neuroprotective properties [22,23,24]. Recent in vitro and in vivo data also demonstrated the positive role of adiponectin signaling in neural stem cell proliferation [18,25,26]. Given that many neuroprotective strategies have failed in human trials (i.e., Citicoline) [27], modulation of adiponectin signaling may be an attractive way to promote neuroprotection.

In addition, the difficulties in producing adiponectin, as well as its rapid turnover, make the use of adiponectin protein for therapeutic benefits laborious and almost outdated [28,29]. To overcome this issue, agonists of adiponectin receptors have been developed such as AdipoRON [30,31]. AdipoRON exhibits interesting features including the reduction in renal inflammation in obese mice and suppression of tumor growth in human pancreatic cancer [32,33,34,35]. In a mouse model of Alzheimer’s disease, AdipoRON improves cognitive impairment and neurological complications [36]. In addition, this drug attenuates neuroinflammation after intracerebral hemorrhage [37]. AdipoRON also has the ability to cross the blood-brain barrier [36].

Very interestingly, the preventive injection of adiponectin in mice and rats before middle cerebral artery occlusion (MCAO) reduced cerebral infarct volumes and improved neurological scores [38,39,40] However, the therapeutic effects of modulating adiponectin signaling in stroke condition are not fully characterized and deserve attention. The purpose of this study was first to further examine the distribution and the identity of AdipoR1/R2-expressing cells within the brain, and then to determine the impact of AdipoRON at the reperfusion in a MCAO model, 24 h post-ischemia. To this aim, we first performed immunohistofluorescence experiments to determine the distribution of AdipoR1 and AdipoR2 within the brains of adult mice. Then, we performed a 90 min MCAO on mice and injected AdipoRON (5 mg.kg^−1^, a dose known to activate both AdipoR1 and R2 and to display therapeutic effects) during the reperfusion phase for analyzing its impact on the infarct size, neuroinflammation and neurological score 24 h post-stroke.

## 2. Materials and Methods

### 2.1. Animals and Ethic

C57BL/6 mice (eight weeks old, male, around 25 g) were purchased from Charles River Laboratory (Saint Germain Nuelles, France). They were maintained under standard conditions of light (12 h light/12 h dark cycle), temperature and humidity with free access to water and standard chow diet. All experiments were conducted in accordance with the French and European community Guidelines for the Use of Animals in Research (86/609/EEC and 2010/63/EU) and approved by the local Ethics Committee for animal experimentation of CYROI (APAFIS#19832-2018092522279654_v8).

### 2.2. Middle Cerebral Artery Occlusion (MCAO) Procedure and AdipoRON Treatment

Brain ischemia/reperfusion was performed as previously described on a total of 20 mice. Briefly, mice were anesthetized with isoflurane (IsoFlo^®^ Centravet, Paris, France) and the body temperature was maintained to 34 °C. The MCAO was conducted for 90 min by introducing a 7-0 silicon rubber-coated monofilament (702056PK5, Scholz Group, Doccol Corporation, Sharon, MA, USA) through the right common carotid at the bifurcation of the right MCA and the right internal carotid. After this 90 min occlusion procedure, the monofilament was removed and reperfusion was performed with random injection of Vehicle or AdipoRON directly in the right common carotid (AdipoRON group: 5 mg.kg^−1^ of body weight versus vehicle saline group for a corresponding volume from 40 to 50 µL according to the body weight).

AdipoRON was used in a preclinical model for different pathologies at doses varying between 0.1 mg.kg^−1^ to 50 mg.kg^−1^. The K_D_ values of AdipoRON are 0.6 µg.mL^−1^ for AdipoR1 and 1.3 µg.mL^−1^ for AdipoR2 [30] and intermediate K_D_ value of globular adiponectin for their receptor was around 0.8 µg.mL^−1^ [41]. Globular adiponectin has a protective role during ischemia at 0.1 mg.kg^−1^ with an injection before ischemia [40] or at 5 mg.kg^−1^ post ischemia [42]. In the light of these data, AdipoRON was used at 5 mg.kg^−1^ in our study, a dose comparable to the effective dose of globular adiponectin in ischemia regarding the relative K_D_ value of these ligands for adiponectin receptors. This dose was also the starting dose for beneficial gastroprotective effects in a recent study [43].

The animals were euthanized 24 h post-ischemia. The brains were carefully and quickly dissected, and processed for further analyses.

### 2.3. Measurement of Infarct Volume

Coronal brain sections (1 mm section-thickness) were performed and incubated with a 2% solution of 2,3,5-triphenyltetrazolium chloride (TTC, Sigma Aldrich, Schnelldorf, Germany) for 20 min at room temperature. The TTC staining is a colorless water-soluble dye reduced to a deep red, water-insoluble compound (formazan) within the mitochondria of living cells [44]. The alive part of the brain appears red while the dead part remains white. The volume of the infarct area was assessed on five brain slices of 1 mm thickness per animal (*n* = 8–10), as previously described [45] using NIH ImageJ software. This procedure was carried out in blind conditions by two independent observers.

### 2.4. Neurological Evaluation

The functional deficit was assessed 24 h after reperfusion using the Bederson scale: scale 0, no deficit; 1, mild forelimb weakness; 2, severe forelimb weakness, consistently spins to the side of deficit when lifted by the tail; 3, mandatory spinning; 4, unconscious; and 5, death [46].

### 2.5. Tissue Preparation

For AdipoR immunohistofluorescence, mice were euthanized and intracardially perfused with 1x-PBS (Phosphate Buffered Saline) containing 4% paraformaldehyde (PBS-PFA, pH 7.4). Then, the dissected brains were fixed for an additional overnight period at 4 °C. For paraffin embedding, brains were dehydrated and processed for paraffin inclusion. Coronal sections (7 µm-thickness) were obtained using a microtome (Microm HM 340E ThermoFisher Scientific, Les Ulis, France). For cryostat embedding, brains were first cryoprotected with 30% sucrose-PBS for 48 h at 4 °C. The brains were next embedded in Tissue-Tek^®^ OCT compound and frozen at –80 °C. Coronal sections (12 µm-thickness) were obtained using a cryostat (Leica CM 15 20, Wetzlar, Germany).

For RNA and protein extraction, the ipsilateral and contralateral hemisphere of the 1 mm coronal section were separated and snap frozen.

### 2.6. RNA Extraction qPCR Analysis

Contralateral and ipsilateral hemispheres were separated and processed for RNA extraction, RT-PCR and quantitative real-time polymerase chain reaction (qPCR) experiments in order to determine the effect of AdipoRON on the expression of several inflammatory genes.

For the qPCR experiments, the AB7500 real-time PCR system (Applied Biosystems, Foster City, CA, USA) was used with the SYBR green master-mix (Eurogentec; Rue du Bois Saint-Jean, Belgium). Specific mouse primers were used (Table 1). In order to confirm the correct amplification, PCR efficiency and melting curves were performed. The CFX software (Biorad) was used to analyze the results. The relative expression of *Il-6*, *Nrf2*, *Nfκβ* and *Tnfa* genes was normalized against the expression level of the housekeeping *Gapdh* gene using the delta-delta Ct method.

### 2.7. Immunohistofluorescence

For AdipoR1 staining, paraffin sections were dewaxed, rehydrated through graded ethanol series (100–30%) and rinsed in 1X PBS. Antigen retrieval was performed using a 10 mM sodium citrate buffer (pH 6) in a microwave (750 W) for 2 min, and slides were maintained for 5 min in the warm buffer at room temperature. Slides were washed twice in PBT (1X PBS containing 0.2% Triton X100). Endogenous peroxidases were inhibited using 0.3% H_2_O_2_ in PBS for 30 min. After washing in PBT, non-specific binding was blocked in PBT containing 2% BSA (Bovine Serum Albumin; Schnelldorf, Germany) for 45 min. Specific primary and secondary antibodies were used (Table 2 and Table 3). Finally, brain sections were incubated with rabbit anti-AdipoR1 (Abcam, Paris, France; REF: ab240022; 1:250) in 0.5% BSA-PBT overnight at room temperature. The next day, the slides were washed several times in PBT and incubated with a donkey anti-rabbit-Horseradish peroxidase (Jackson laboratories; REF: 711-035-152; 1:1000) for 1 h 30. Then, after being washed with PBT, staining was performed using tyramide signal amplification (Alexa Fluor 594; ThermoFischer, Les Ulis, France; REF: B40957; 1:200) according to the manufacturer’s recommendations with DAPI nuclear counterstaining.

For AdipoR2 staining, cryostat sections were rehydrated in PBS. Antigen retrieval was performed, as described above. After washing in PBT, non-specific binding was blocked in PBT containing 2% BSA for 1 h. Finally, brain sections were incubated with rabbit anti-AdipoR2 (LifeSpan BioSciences, Bergerden, The Netherlands; REF: LS-C34900; 1:250) in 0.5% BSA-PBT overnight at room temperature. The next day, slides were washed in PBT and incubated for 1 h 30 m with secondary antibodies Alexa fluor 594 (Abcam, Les Ulis, France; REF: ab150064; 1:500) and DAPI for cell nuclear counterstaining.

For the co-labelling studies of AdipoR1 and AdipoR2 with markers of neurons, microglia and astrocytes, incubation was also performed with antibodies specific to these different cell types, respectively, anti-HuC/D (ThermoFisher, Les Ulis, France; REF: A21271, clone AB11; 1:100), anti-Iba1 (Abcam, Paris, France; REF: ab5076; 1:200; allograft inflammatory factor 1), and anti-GFAP (Abcam, Paris; France; REF: ab53554, 1:500; glial fibrillary acidic protein).

Incubation without primary antibody or with non-relevant IgG resulted in the absence of any staining (even with the TSA system) (Supplementary Materials Figure S1). The specificity of the staining was also reinforced by *AdipoR* in situ hybridization coupled to HuC/D immunostaining (Supplementary Materials Figure S1), as previously described by our laboratory [18]. The results showed in this study have been performed in at least three different brains.

For cleaved-caspase 3 and Iba1 staining in MCAO brains, cryostat sections were rehydrated in PBS. After washing in PBT, non-specific binding was blocked in PBT containing 2% BSA for 1 h and incubation was made overnight with primary antibodies. The next day, slides were washed in PBT, incubated for 1 h 30 m with secondary antibodies Alexa fluor 488 (Abcam, Paris; France; REF: ab150061; or ThermoFisher, Les Ulis, France; A11055; 1:500) and DAPI for cell nuclear counterstaining.

### 2.8. Western Blot

Frozen contralateral and ispsilateral hemispheres from control and AdipoRON-treated mice were homogenized with a tissue lyser (Qiagen; Courtaboeuf, Cedex, France) in lysis buffer (50 mM Tris-HCl pH 9, 5 mM EDTA, 500 mM NaCl) containing a mixture of protease and phosphatase inhibitors (Pierce, Thermofischer, Waltham, MA, USA). Total protein extract concentration was evaluated using BCA assay (Sigma-Aldrich, St. Louis, MO, USA). Twenty micrograms of proteins were separated on 16% SDS polyacrylamide gel and transferred to nitrocellulose, followed by immunoblotting as described before [47]. The incubation was performed with specific antibodies: mouse anti-*Nrf2* (Abcam, Les Ulis, France; REF: ab31163, 1:1000) at 4 °C overnight. The following day, membranes were washed and incubated with goat HRP-conjugated secondary antibodies (Jackson IR, Cambridge, UK; REF: 111-035-003, 1:1000) for 1 h 30 m before enhanced chemioluminescence detection, as recommended by the manufacturer with Amersham Imager 680.

### 2.9. ELISA Assay

TNF⍺ levels were measured as previously described [47] in contralateral and ipsilateral hemispheres of control and AdipoRON-treated mice. Briefly, in order to quantify TNF⍺ in our samples, the capture antibodies anti-TNF⍺ (eBioscience, Dardilly, France; REF: 14-7423-68,1:250) were coated in a 96-well plate overnight at room temperature. The following day, samples were added in each well at 1:10 in diluent solution. For the detection step, the biotinylated detection antibodies mouse anti-TNF⍺ (eBioscience, Dardilly, France; REF: 13-7341-68A, 1:250) were used for 1 h at room temperature. After this period of time, streptavidin-HRP (eBioscience, Dardilly, France; REF: 00-4100-94, 1:250) was added.

### 2.10. Microscopy

An Eclipse 80i Nikon microscope equipped with a Hamamatsu digital camera (Life Sciences, Tokyo, Japan), a nanozoomer S60 (Hamamatsu) and a laser scanning confocal microscope Eclipse confocal (Nikon, Tokyo, Japan) were used. Pictures were adjusted for brightness and contrast in Adobe Photoshop CS7.

### 2.11. Statistical Analysis

Comparisons between two groups were performed using a statistical Student’s *t*-test. Comparisons between more than two groups were performed using ANOVA multiple testing. All error bars correspond to the SEM, and *n* values correspond to the number of animals, *p* < 0.05 was considered as statistically significant; * *p* < 0.05; ** *p* < 0.01 and *** *p* < 0.001.

## 3. Results

### 3.1. Adiponectin Receptors Are Widely Distributed in the Brain of Adult Mice

**Figure 1:**
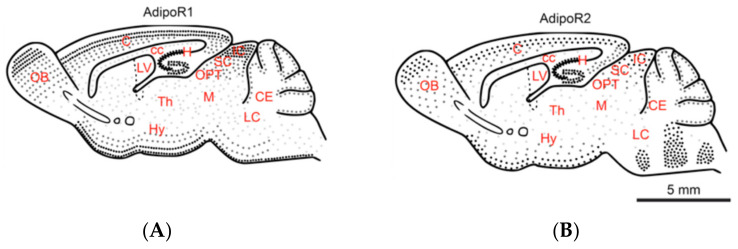
Schematic representation of AdipoR1 and AdipoR2 distribution on a sagittal mouse brain section. (**A**) AdipoR1 and (**B**) AdipoR2 are widely expressed throughout the main brain subdivisions and display overlapping patterns. Each black dot represents AdipoR1 (**A**) and AdipoR2 (**B**) staining. C: Cortex, cc: corpus callosum, CE: Cerebellum H: Hippocampus, Hy: Hypothalamus, IC: Inferior colliculus, LC: Locus coeruleus, LV: Lateral ventricle, M: Mesencephalic tegment, OB: Olfactory bulbs, OPT: Olivary pretectal nucleus, SC: Superior colliculus, Th: Thalamus. Scale bar: 5 mm.

Adiponectin signaling in the CNS has been studied in the last few years. However, the data concerning adiponectin receptor distribution (AdipoR1 and AdipoR2) in the CNS are only partial and come from studies performed in different species and from different developmental/adulthood stages. Consequently, we first evaluated the overall expression and distribution of both receptors by performing AdipoR1 and AdipoR2 immunohistostaining in the brains of eight-week-old adult male mice. Then, the different AdipoR1 and AdipoR2-positive cells were documented in a sagittal mouse brain scheme (Figure 1).

AdipoR1 and AdipoR2 were widely expressed in the brain, comforting previous ISH and RNA sequencing data [17,18]. The specificity of the staining was comforted by the absence of labeling with incubation in absence of primary antibodies or with non-relevant IgG (Supplementary Materials Figure S1). AdipoR1 was expressed in all brain regions (tel-, di-, mes-, met- and myel-encephalon), including the olfactory bulbs (OB), the cerebral cortex (C), the thalamus (Th), the hypothalamus (Hy), the mesencephalic tegmentum (M), the cerebellum (CE) and the pons. AdipoR1 was also detected in the whole striatum and in neurogenic regions such as the hippocampus and the subventricular zone of the lateral ventricle (LV). In some brain regions such as in the cerebral cortex, AdipoR1 expression appeared to be regionalized. For instance, the outer cerebral cortical layer was shown to be composed of a low number of AdipoR1-positive cells while the other cortical layers were more densely stained.

For AdipoR2, immunohistostaining demonstrated a large expression in the main brain subdivisions (tel-, di-, mes-, met and myel-encephalon) with numerous AdipoR2-positive cells detected in the olfactory bulbs (OB), the cerebral cortex (C), the striatum, the thalamus (Th), the hypothalamus (Hy), the mesencephalic tegmentum (M), the cerebellum (CE). In addition, AdipoR2-positive cells were also detected in the hippocampus, as well as in the ventral periphery of the brain. AdipoR2 staining was also observed in the pons (i.e., the reticular formation and the inferior olive). As for AdipoR1, AdipoR2 expression appeared somehow regionalized. Indeed, a lower number of AdipoR2-positive cells were reported in the striatum, the mesencephalic tegmentum, the pons and the cerebellum.

Together, our data showed a wide overlapping distribution of AdipoR1 and AdipoR2-positive cells within the brain, with a substantially stronger staining for AdipoR1.

### 3.2. Adiponectin Receptors Are Mainly Expressed by Neurons

**Figure 2:**
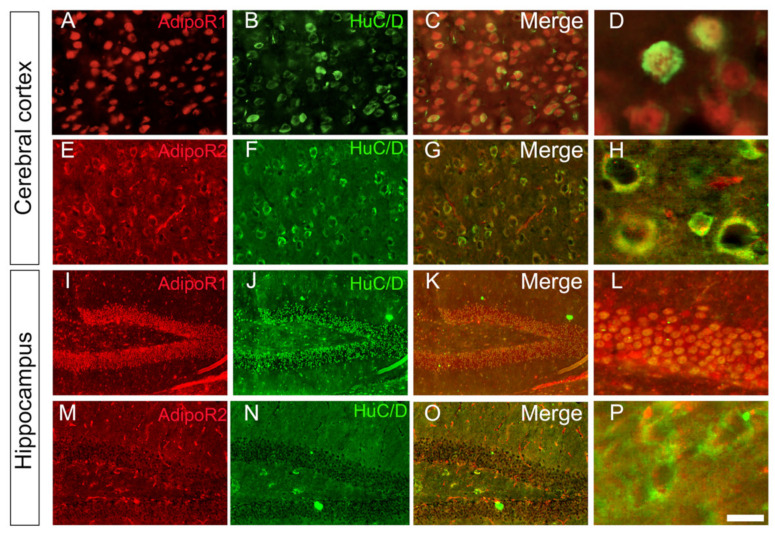
AdipoR1 and AdipoR2 are expressed by neurons in the cortex and hippocampus of mice. (**A**) AdipoR1 and (**E**) AdipoR2 immunostaining (red) with HuC/D neuronal co-labelling (green, (**B**,**F**)) in the cerebral cortex demonstrating major co-staining (yellow, (**C**,**D**,**G**,**H**)). (**I**) AdipoR1 and AdipoR2 (**M**) immunostaining (red) with HuC/D neuronal co-labelling (green, (**J**,**N**)) in the dentate gyrus of the hippocampus. AdipoR1 is strongly expressed in neurons, as shown in the merge pictures (yellow, (**K**,**L**)). Although AdipoR2 appears less expressed in the hippocampus, it co-localized with the neuronal marker (yellow, (**O**,**P**)). Note that AdipoR2 is also detected in structures looking like blood vessels. (**D**,**H**,**L**,**P**)**:** Higher magnifications. Scale bar: 7 µm (**D**,**H**), 14 µm (**L**,**P**), 200 µm (**A**–**G**) and 400 µm (**I**–**O**).

In order to determine the identity of brain cells expressing AdipoR, co-labelling of AdipoR1 and R2 was performed with neuronal (HuC/D), microglial (Iba1) and astrocytic (GFAP) markers. We focused on two main brain regions where both receptors were expressed: the cortex and the hippocampus. As shown in Figure 2, AdipoR1 and AdipoR2 were mainly expressed in HuC/D-positive neurons in the cerebral cortex and in the hippocampus. However, the levels of AdipoR2 expression in the dentate gyrus appeared weaker than for AdipoR1. In the other brain regions, these co-expressions were also largely observed. These data were corroborated by the fact *AdipoR1* and *AdipoR2* in situ hybridization coupled to HuC/D immunohistochemistry demonstrated co-expression of cells expressing *AdipoR* transcripts with neurons (Supplementary Materials Figure S1). These results were previously documented by Rastegar et al. (2019) [18].

**Figure 3:**
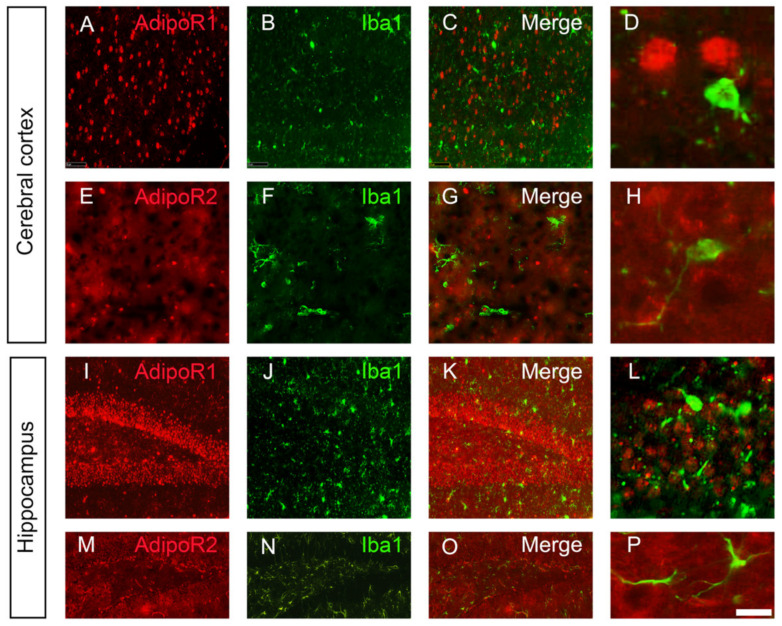
AdipoR1 and AdipoR2 are not strongly expressed by the microglia in the cerebral cortex and hippocampus. (**A**) AdipoR1 and (**E**) AdipoR2 immunohistostaining (red) with Iba1 microglial co-labelling (green, (**B**,**F**)) in the cerebral cortex showing no co-expression (**C**,**D**,**G**,**H**). (**I**) AdipoR1 and (**M**) AdipoR2 immunostaining (red) with Iba1 microglial co-labelling (green, (**J**,**N**)) in the dentate gyrus of the hippocampus showing no co-expression (**K**,**L**,**O**,**P**). (**D**,**H**,**L**,**P**): Higher magnifications. Scale bar = 7 µm (**D**,**H**), 14 µm (**L**,**P**), 200 µm (**A**–**O**).

**Figure 4:**
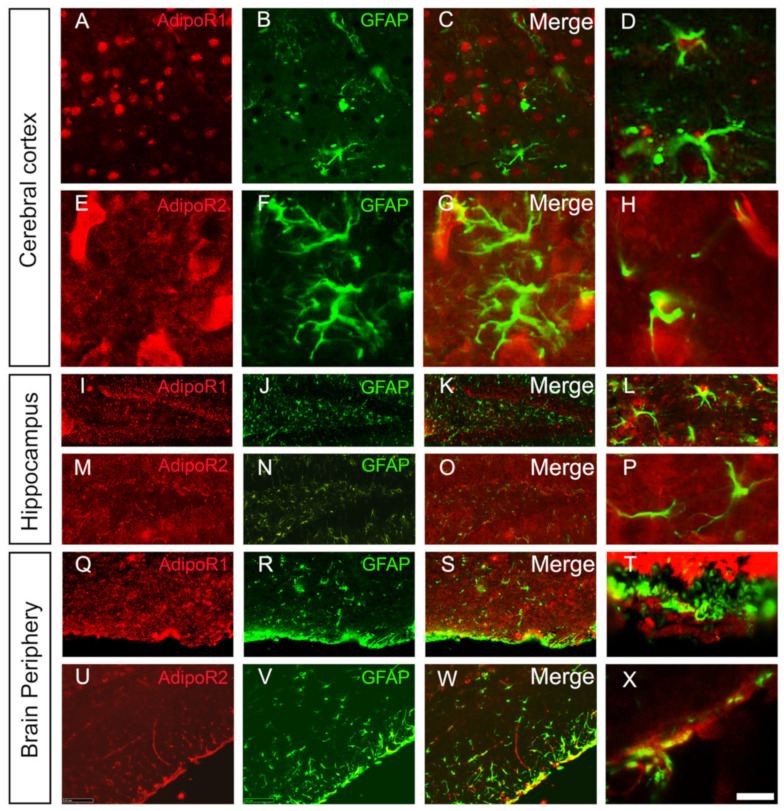
AdipoR1 and AdipoR2 are not strongly expressed by astrocytes but AdipoR2 is detected in some astrocytic end-feet in the ventral part of hypothalamus. (**A**) AdipoR1 and (**E**) AdipoR2 immunostaining (red) with GFAP astrocyte co-labelling (green, (**B**,**F**)) in the cerebral cortex demonstrating no obvious co-staining (**C**,**D**,**G**,**H**). (**I**) AdipoR1 and (**M**) AdipoR2 immunostaining (red) with GFAP astrocyte co-labelling (green, (**J**,**N**)) in the dentate gyrus of the hippocampus demonstrating no obvious co-staining (**K**,**L**,**O**,**P**). (**Q**) AdipoR1 and (**U**) AdipoR2 immunostaining (red) with GFAP astrocyte co-labelling (green, (**R**,**V**)) in the brain periphery demonstrating AdipoR2 staining in some astrocyte end-feet (yellow, (**W**,**X**)) in contrast to AdipoR1 (**S**,**T**). (**D**,**H**,**L**,**P**,**T**,**X**)**:** Higher magnifications. Scale bar = 7 µm (**A**–**H**) and (**L**–**P**), 100 µm (**I**–**W**), 200 µm (**T**–**X**).

Co-labelling with the microglial and astrocytic markers (Iba1 and GFAP, respectively) demonstrated that microglia and astrocytes did not significantly express AdipoR1 and AdipoR2 in all the brain regions studied (Figure 3 and Figure 4). This was previously shown for astrocytes by Rastegar et al. (2019) [18]. Nevertheless, at the ventral brain periphery, numerous astrocyte end-feet appeared to be AdipoR2-positive (Figure 4).

**Figure 5:**
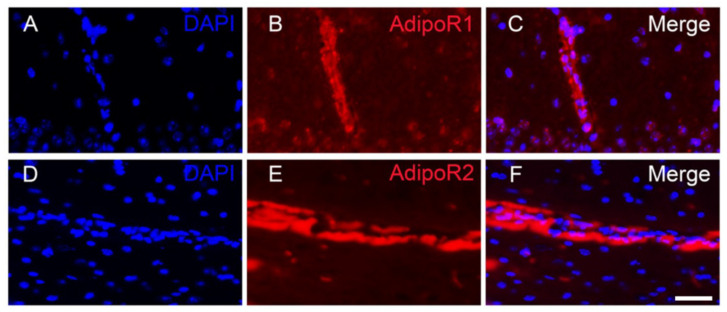
AdipoR1 and AdipoR2 are expressed in blood vessels. (**A**–**F**). AdipoR1 and AdipoR2 immunostaining (red) delimits blood vessel-like structures. In blue: DAPI counterstaining. Scale bar = 40 µm.

Taken together, this neuroanatomical study demonstrated that AdipoR1 and AdipoR2 are expressed throughout the main subdivisions of the brain. Both receptors display an overlapping distribution and are mainly expressed in neurons. However, AdipoR2 was also detected in some astrocyte end-feet (Figure 4). Interestingly, we also observed AdipoR1 and AdipoR2 staining in blood vessels, probably in endothelial cells (Figure 5). In addition, the specificity of the staining was reinforced by convergent data between AdipoR in situ hybridization and AdipoR immunostainings (with obvious expression of AdipoR transcripts and proteins in neurons, but not mainly in astrocytes). Given that brain ischemia induced by the MCAO procedure mainly affects the cerebral cortex and the striatum, in which AdipoR are expressed, we decided to further investigate the potent therapeutic effect of modulation of adiponectin signaling during brain ischemia.

### 3.3. AdipoRON Treatment Has No Therapeutical Effect on the Infarct Size

**Figure 6:**
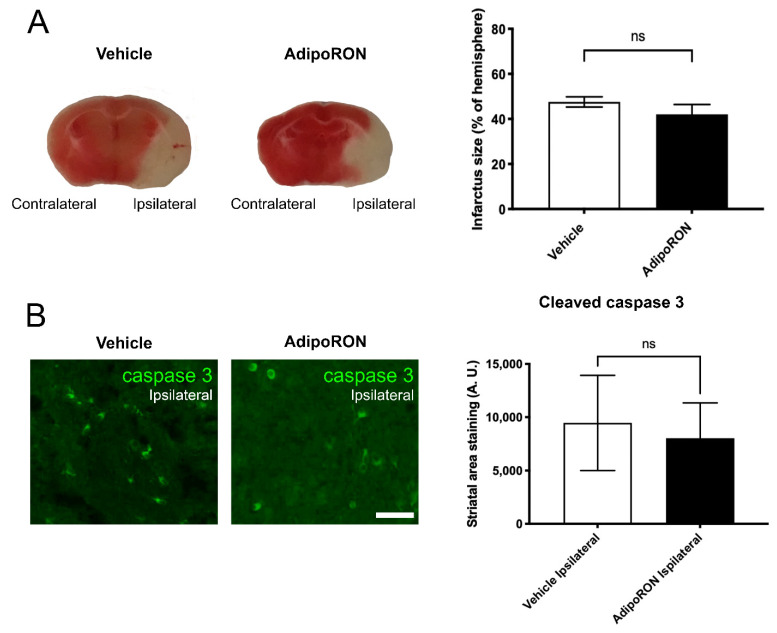
AdipoRON has no effect on the infarct size and on the number of activated caspase 3-positive cells 24 h post-stroke. (**A**) Representative coronal brain sections stained with TTC (ischemic/necrotic brain region is in white, and the alive region is in red) showing the contralateral and ipsilateral hemispheres. Quantification of the size of the infarcted area showing no difference between groups (*n* = 8–10, *p* = 0.3158). (**B**) Representative immunohistostaining of activated caspase 3 (green) in the ipsilateral hemisphere of Vehicle and AdipoRON-treated mice. Quantification of cleaved caspase 3 staining in the striatal area of the ipsilateral hemisphere showing no significant difference between groups (*n* = 4, *p* = 0.63). Scale bar = 40 µm (**B**) and 200 µm (**A**).

To investigate the role of adiponectin signaling in brain ischemia, we made use of the adiponectin receptor agonist AdipoRON. After a 90 min MCAO procedure, AdipoRON (5 mg.kg^−1^) was injected into the mice during the reperfusion step. The infarct size and neuroinflammation were analyzed 24 h post-stroke. After TTC staining of brain sections (Figure 6A), the quantification of the infarct size demonstrated that AdipoRON treatment did not impact the damage area compared to the vehicle group (Infarct size: 47% of the hemisphere ± 2.2 vs. 42% ± 4.4 in vehicle and AdipoRON group, respectively).

To further confirm the absence of effect of AdipoRON treatment on cell death, we monitored apoptosis in mouse brain sections using immunostaining for the cleaved active form of caspase 3. In the ipsilateral hemisphere of vehicle and AdipoRON-treated mice, no difference in caspase 3 staining was observed in the striatal area (Figure 6B), reinforcing the TTC results. In addition, the neurological score was similar between Vehicle- and AdipoRON-treated mice (3.68 ± 0.13 vs. 3.5 ± 0.22, respectively; *p* > 0.5 for *n* = 8–10). Consequently, in our experimental conditions, AdipoRON had no significant effect on cell death at 24 h post-stroke and on the neurological outcomes.

**Figure 7:**
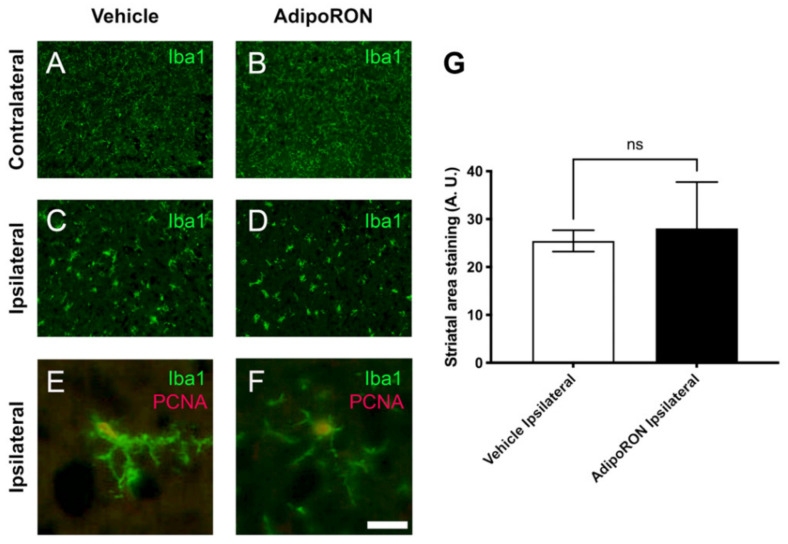
AdipoRON has no effect on the infarct size and on the number of activated caspase 3-positive cells 24 h post-stroke. (**A**–**D**) Iba1 immunostaining (green) in the contralateral (**A**,**B**) and ipsilateral (**C**,**D**) hemispheres of Vehicle and AdipoRON-injected mice. (**E**,**F**) Iba1 and PCNA co-stainings showing proliferation of microglia in the ipsilateral hemisphere of Vehicle and AdipoRON-treated mice (yellow). (**G**) Quantification of Iba1-positive staining in the striatal area of the ipsilateral hemispheres showing no difference between both groups. A.U. = Arbitrary Unit. Scale bar = 7 µm (**E**,**F**), 200 µm (**A**–**D**).

Considering the roles of Adiponectin-AdipoR axis in inflammatory processes, we decided to investigate the recruitment and activation of microglial cells. These cells are a major source of pro-inflammatory factor secretion and their amoeboid morphology is a reflection of their activation and inflammatory state. For this purpose, microglia were studied within the damage area by Iba1 immunostaining (Figure 7). In the contralateral hemisphere, Iba1-positive cells were widely found in a resting state (small cell body and densely ramified) in control and AdipoRON-treated conditions (Figure 7A,B). In contrast, in the ipsilateral hemisphere of each group, microglial cells were hypertrophic, less ramified and displayed an activated/amoeboid shape (Figure 7C,D). As expected, microglial cells were highly proliferative in the ipsilateral hemispheres and displayed a stronger Iba1 staining (Figure 7E,F). These features are characteristic of microglial activation. As expected, the quantification of Iba1 immunostaining showed that brain ischemia resulted in a significant increase in Iba1-positive labelling in the ipsilateral hemisphere compared to the contralateral one, in both Vehicle and AdipoRON-treated mice (data not shown). However, no difference was observed in microglia reactivity in the ipsilateral hemispheres of control mice compared to AdipoRON-treated ones (Figure 7G).

**Figure 8:**
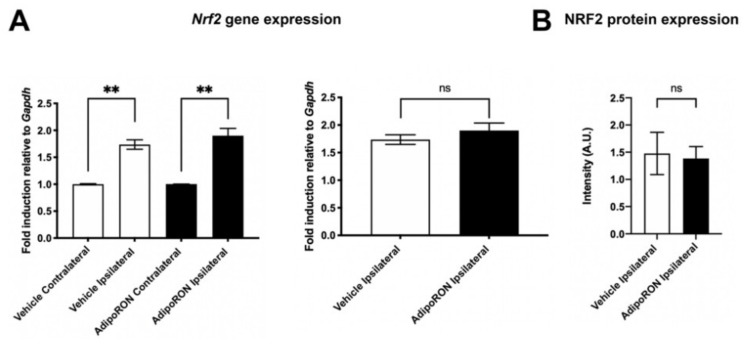
AdipoRON did not change the gene and protein expression of Nrf2 in the ischemic hemisphere compared to control mice. (**A**) *Nrf2* gene expression is up-regulated in the ipsilateral hemisphere compared to the contralateral one in both Vehicle and AdipoRON-treated group. Note that *Nrf2* gene expression remains similar in the ipsilateral hemisphere of both groups. (**B**) NRF2 protein expression in the ipsilateral hemisphere is unchanged 24 h post-stroke in Vehicle and AdipoRON-treated mice (*n* = 3). ** *p* < 0.01.

In parallel, our qPCR analyses showed a quantitative increase in transcripts for *Nrf2*, a transcriptional factor known to be up-regulated by oxidative stress and to play a key role in the maintenance of redox homeostasis and in the regulation of inflammation. Indeed, *Nrf2* was increased in the ipsilateral hemisphere compared to the contralateral one, in both control and treated conditions (×1.7 and ×1.9, respectively; *p* < 0.01). However, there was no significant difference in *Nrf2* gene expression and in NRF2 protein levels in the ipsilateral hemispheres of Vehicle and AdipoRON-treated mice (Figure 8).

**Figure 9:**
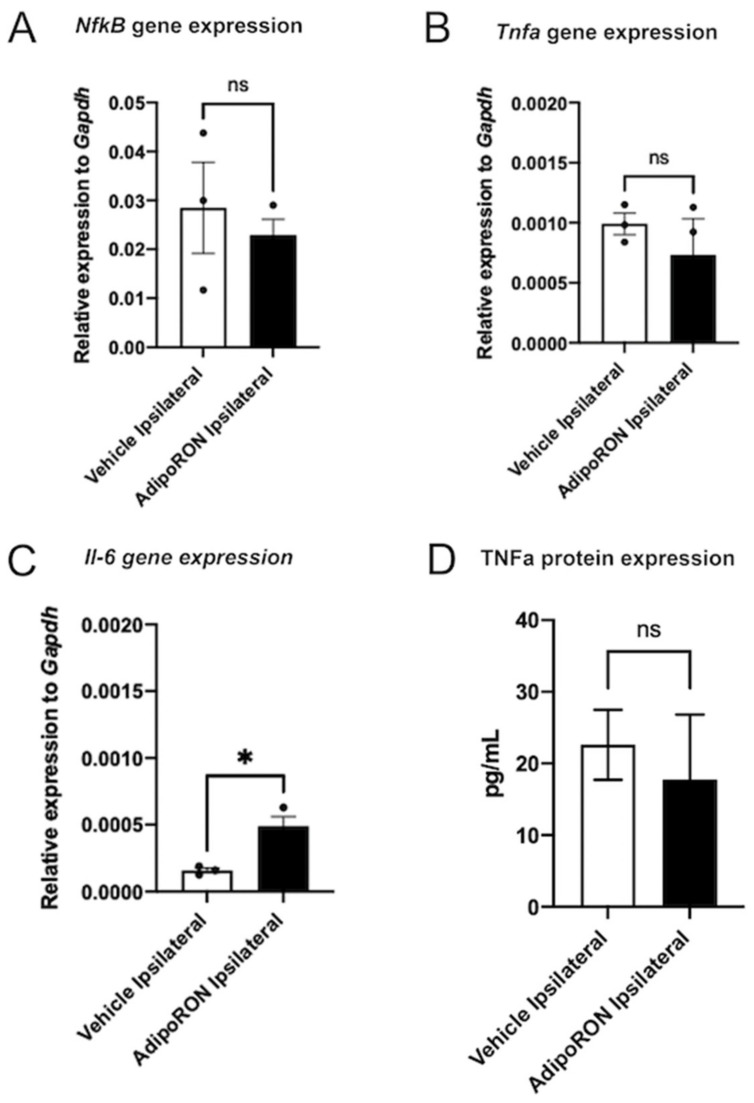
AdipoRON has no effect on inflammatory cytokine expression in the ipsilateral hemisphere. (**A–C**) *Nfκβ*, *Il-6* and *Tnf⍺* gene expression in the ipsilateral hemispheres of Vehicle and AdipoRON-treated groups. (**D**) TNF⍺ ELISA quantification showing no difference in the ipsilateral hemispheres of both groups (*n* = 3). * *p* < 0.05.

We then analyzed the expression levels of *Nfκβ* (a transcription factor known to be involved in the inflammatory process) and of pro-inflammatory cytokines (*Tnfɑ* and *Il-6*) in the ischemic hemispheres of Vehicle and AdipoRON-treated mice. The qPCR analysis revealed no significant difference in *NfκB* and *Tnf⍺* levels between Vehicle and AdipoRON-treated groups (Figure 9A,B). This was also confirmed by ELISA assay, showing no difference in TNF⍺ secretion in the ipsilateral hemispheres (Figure 9D). Surprisingly, *Il-6* gene expression was significantly higher in the ischemic hemispheres of AdipoRON-treated mice compared to vehicle group (Figure 9C).

Taken together, these data indicate that, under the conditions being assessed, AdipoRON had no impact on the infarct size, microglial recruitment or neuro-inflammatory processes.

## 4. Discussion

The present study highlighted the overlapping and wide distribution of adiponectin receptors in the brain of adult mice, demonstrating that AdipoR are expressed by neurons. This expression suggests that the brain and neural tissue are well able to respond to adiponectin signaling. We thus tested the hypothesis of a neuroprotective signaling of AdipoRON in stroke. However, we showed in a model of brain ischemia (MCAO) that a single injection of AdipoRON (5 mg.kg^−1^ of body weight) during the reperfusion step had no impact on the size of the ischemic area, as well as on apoptotic and neuro-inflammatory processes 24 h post-stroke.

### 4.1. Adiponectin Receptors Are Widely Expressed throughout the Brain

This work provides an important insight into the overall general distribution of AdipoR1 and AdipoR2 in the brain of adult mice. The results are in agreement with previous studies documenting AdipoR expression in the cortex, the hypothalamus, the pituitary gland, the brainstem and the hippocampus and reveals a stronger expression compared to other brain areas, as reviewed by [1]. We also demonstrated the co-expression of both AdipoR with HuC/D-positive neurons, namely in the cortex and the hippocampus. This is in accordance with in situ hybridization data and other immunostaining studies performed in rodents [18,48]. We did not observe any striking co-labelling of AdipoR with the Iba1 microglial marker, while some in vivo and in vitro studies show that murine microglia express both AdipoR [1,22,49]. Similarly, we did not observe prominent co-expression with astrocytes, which is consistent with our previous *AdipoR* in situ hybridization [18]. However, AdipoR2 staining was detected in some astrocyte end-feet of the mediobasal hypothalamus. AdipoR1 and AdipoR2 expression was also detected in the wall blood vessels, in cells resembling to endothelial cells as previously described [50,51]. In other studies performed in the spinal cord, AdipoR expression was observed in neurons, astrocytes and microglia [52]. These small discrepancies between our results and the heterogeneous data from the literature could be due to heterogeneity in rodent models used (mice vs. rat), age and sex of the animals, regions studied (brain vs. spinal cord), as well as different set of antibodies used. The conclusion of our work remains that the brain is a major target tissue for adiponectin signaling.

### 4.2. Adiponectin Signaling as a Therapeutical Target for Stroke?

Considering the pleiotropic effects of adiponectin (i.e., anti-inflammatory, antioxidant, pro-survival), the modulation of adiponectin signaling during brain ischemia could be an interesting way to limit brain damage and promote brain repair. Previous studies have shown that two preventive injections of adiponectin (from 5 mg.kg^−1^) before the MCAO surgery significantly improved the neurological score correlated to a reduced infarct size and oxidative stress [23,53]. Additionally, an injection of adiponectin (5 µg, 30 min before MCAO and immediately after) was able to significantly down-regulate pro-inflammatory cytokines (i.e., TNF⍺, Il-1β) and NFκB protein levels 24 h post-stroke in rats [38,54]. In another study, Zhang and colleagues showed that repeated injections of adiponectin (5 mg.kg^−1^) (6 h, 24 h and 48 h post-stroke) decreased the ischemic volume, oxidative stress and apoptosis and improved the neurological score compared to control mice [42]. So far, adiponectin has shown preventive and therapeutic effects on the stroke outcomes in preclinical studies.

In our study, we report that a single injection of AdipoRON (5 mg.kg^−1^, a dose known to activate both AdipoR, and to exhibit gastroprotective effects [43]) did not limit the infarct size and apoptosis 24 h after stroke. In addition, *Nrf2* gene expression known to be up-regulated after oxidative stress conditions, was significantly higher in the ischemic versus contralateral hemisphere. However, no difference in NRF2 gene and protein expression was observed between the ipsilateral hemispheres of both groups.

In the same line of evidence, in our experimental conditions the level of pro-inflammatory factors remained almost similar in the ipsilateral hemispheres of AdipoRON treated mice compared to the ipsilateral hemispheres of control mice (with the surprising exception of *Il-6*, being upregulated under AdipoRON treatment). These results are also reinforced by a similar activation of microglia, the main neuroinflammatory cells of the brain, in the ischemic hemispheres of both groups.

Interestingly, the daily therapeutic injection of AdipoRON at 50 mg.kg^−1^ during three days post intracerebral hemorrhage (a subtype of stroke) was shown to decrease cell death, oxidative stress and motor impairment in mice [55]. The concentration used, the number of AdipoRON injections, as well as the kinetic time point studied, could be at the origin of the differences observed. It is also possible that the type of stroke performed could lead to differential regulation of AdipoRON efficiency. Furthermore, in other CNS pathologies, AdipoRON (50 mg.kg^−1^) prevents the behavioral defects, the impaired neurogenesis and neuronal death observed in APP/PS1 Tg Alzheimer mice [56]. The anti-inflammatory and anti-oxidative properties of AdipoRON were also observed in other models (diabetes and gastric ulcers) demonstrating the efficiency of this compound [43,57]. AdipoRON could also have an interesting impact on brain plasticity through the modulation of neurogenesis and trophic factors such as brain-derived neurotrophic factor (BDNF) [58].

Taken together, at 24 h post-stroke, our data show no significant therapeutic interest for a single injection of AdipoRON (5 mg.kg^−1^) at the reperfusion step. Recent literature suggests that multiple injections of higher concentrations of AdipoRON may promote brain repair and limit neurological impairment after stroke [37,55]. It is therefore essential to further investigate the effects of such AdipoRON experimental procedures after stroke onset studying: (1) micro- and astrogliosis; (2) the glial scar formation; (3) injury-induced neurogenesis. Moreover, it would be interesting to further investigate the impact of AdipoRON on the different subtypes of ischemic stroke. Indeed, the pathophysiology and clinic of small ischemic vessels are different from those of large infarcts [59]. Considering that transient ischemic attack (TIA) is associated with a better outcome in nonlacunar ischemic stroke, probably by an ischemic tolerance phenomenon, it would be interesting to test the efficacy of AdipoRON on stroke after TIA [60].

Furthermore, although many therapeutic strategies for neuroprotection have failed in human trials, modulation of adiponectin signaling may be an attractive way to promote neuroprotection in humans with high AdipoRON concentrations and multiple injection procedures.

## Figures and Tables

**Table 1:** Mouse qPCR primers.

Gene	Forward	Reverse	Ensembl ID
*Il-6*	CAACAGACTTCCATCCAGTTGC	TTGCCGAGTAGATCTCAAAGTGAC	ENSMUSG00000025746
*Nfκβ*	GTGATGGGCCTTCACACACA	CATTTGAACACTGCTTTGACT	ENSMUSG00000030595
*Nrf2*	TCCCATTTGTAGATGACCATGAG	CCATGTCCTGCTCTATGCTG	ENSMUSG00000015839
*Tnfa*	GTTCTGTCTACTGAACTTCGGG	CAGGCTTGTCACTCGAATTTTG	ENSMUSG00000024401
*Gapdh*	CTTTGTCAAGCTCATTTCCTGG	TCTTGCTCAGTGTCCTTGC	ENSMUSG00000020932

**Table 2:** Primary antibodies.

Antibodies (Marker)	Host	Type	Reference	RRID
AdipoR1	Rabbit	Monoclonal	ab240022	AB_2221906
AdipoR2	Rabbit	Polyclonal	LS-C34900	AB_2222064
GFAP (astrocyte)	Goat	Polyclonal	ab53554	AB_880202
Iba1 (microglia)	Goat	Polyclonal	ab5076	AB_2224402
HuC/D (neuron)	Mouse	Monoclonal	A-21271 Clone 16A11	AB_221448
Cleaved-Caspase 3 (apoptose)	Rabbit	Polyclonal	ab13847	AB_443014
PCNA (proliferation)	Mouse	Monoclonal	MO879 Clone PC10	AB_2160651
NRF2	Host	Polyclonal	ab31163	AB_881705

**Table 3:** Secondary antibodies.

Antibodies	Reference	RRID
Donkey anti-goat Alexa Fluor 488	A11055 (ThermoFisher)	AB_2534102
Donkey anti-mouse Alexa Fluor 488	ab150105 (Abcam)	AB_2732856
Donkey anti-rabbit Alexa Fluor 594	ab150064 (Abcam)	AB_2734146
Donkey anti-rabbit Alexa Fluor 488	ab150061 (Abcam)	AB_2571722
Donkey anti-rabbit-HRP	711-035-152 (Jackson IR)	AB_10015282

## Supplementary Materials

The following supporting information can be downloaded at: https://www.mdpi.com/article/10.3390/brainsci12050680/s1, Figure S1: 1: AdipoR immunostaining controls and AdipoR in situ hybridization. Upper panels: AdipoR1 (A) and AdipoR2 (B) immunostainings in the cerebral cortex showing staining in numerous cells looking like neurons. The negative control (here incubation with non-relevant IgG) shows the absence of staining although incubation with secondary antibodies was done. The same absence of staining was also observed with the incubation without primary antibody (not shown). Lower panels: AdipoR1 (C) and AdipoR2 (D) in situ hybridization in the cerebral cortex coupled with HuC/D immunostaining (neuronal marker) demonstrating the wide expression of transcripts in neuronal cells, in a way similar to what was observed with AdipoR1 and R2 immunostainings (Figure 2). Scale bar = 50 µm (A,B′), 14 µm (C,D).
