# Supplementary material for: Distribution of Adiponectin Receptors in the Brain of Adult Mouse: Effect of a Single Dose of the Adiponectin Receptor Agonist, AdipoRON, on Ischemic Stroke
Source: Brain Sci. 2022 May 23;12(5):680. doi: 10.3390/brainsci12050680 (PMC9139333; doi:10.3390/brainsci12050680)
Supplement: Supplementary file 1 [file brainsci-12-00680-s001.zip › brainsci-1693118-supplementary.pdf]

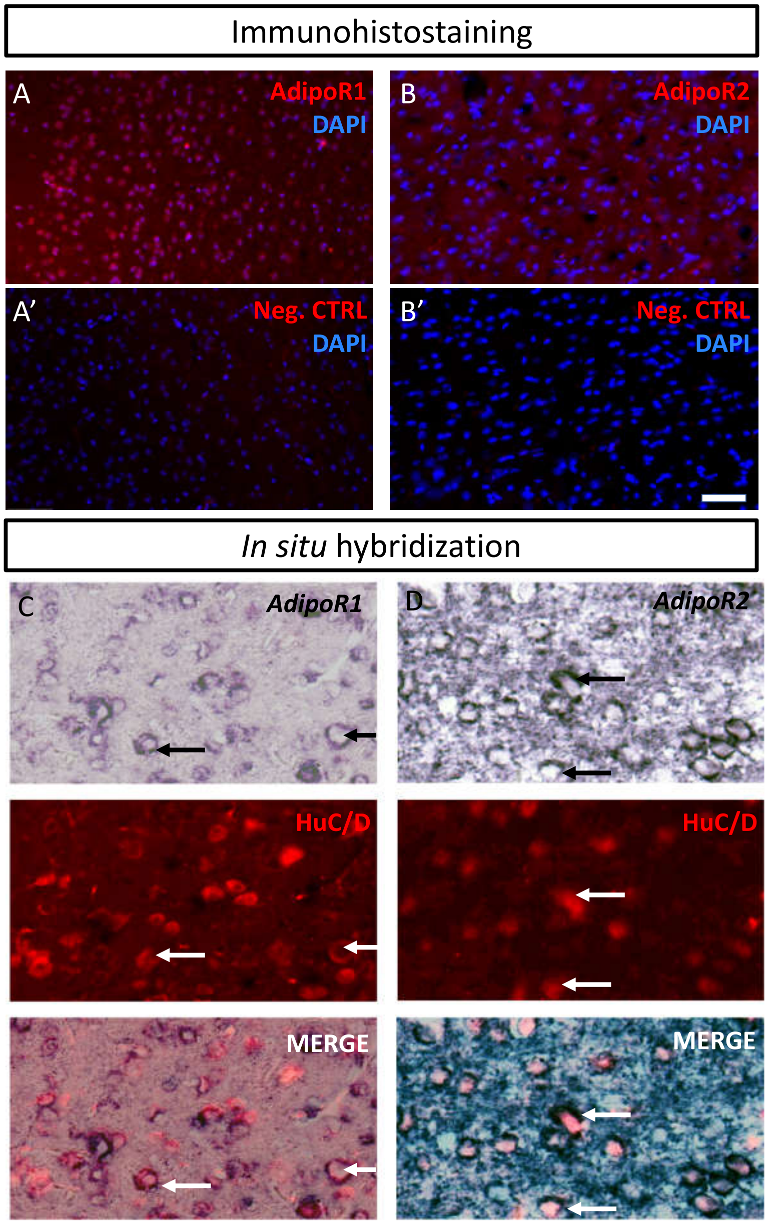

Figure S1. AdipoR immunostaining controls and *AdipoR in situ* hybridization. Upper panels: AdipoR1 **(A)** and AdipoR2 **(B)** immunostainings in the cerebral cortex showing staining in numerous cells looking like neurons. The negative control (here incubation with non-relevant IgG) shows the absence of staining although incubation with secondary antibodies was done. The same absence of staining was also observed with the incubation without primary antibody (not shown). Lower panels: *AdipoR1* **(C)** and *AdipoR2* **(D)** *in situ* hybridization in the cerebral cortex coupled with HuC/D immunostaining (neuronal marker) demonstrating the wide expression of transcripts in neuronal cells, in a way similar to what was observed with AdipoR1 and R2 immunostainings (Figure 2). Scale bar = 50 μm (A-B′), 14 μm (C-D).
